# Supplementary material for: Imputation of spatially-resolved transcriptomes by graph-regularized tensor completion
Source: PLoS Comput Biol. 2021 Apr 7;17(4):e1008218. doi: 10.1371/journal.pcbi.1008218 (PMC8055040; doi:10.1371/journal.pcbi.1008218)
Supplement: S1 Fig — The H&E images are shown on the left, and the heatmaps of the total RNA count at each spot are shown on the right. The regions with irregularly low RNA count are annotated by the circles. (PDF) [file pcbi.1008218.s001.pdf]

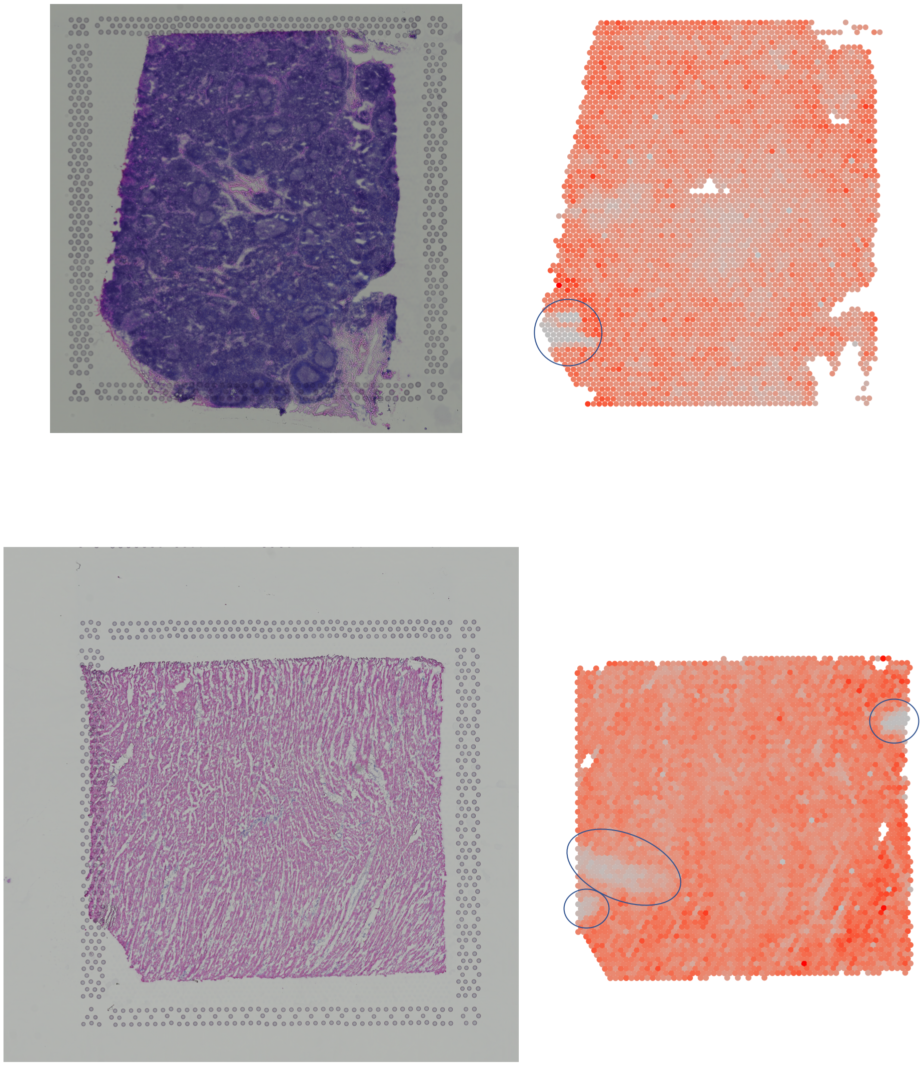

Spatial regions with failed RNA fixing and permeabilization. The H&E images are shown on the left, and the heatmaps of the total RNA count at each spot are shown on the right. The regions with irregularly low RNA count are annotated by the circles.
