# Supplementary material for: Imputation of spatially-resolved transcriptomes by graph-regularized tensor completion
Source: PLoS Comput Biol. 2021 Apr 7;17(4):e1008218. doi: 10.1371/journal.pcbi.1008218 (PMC8055040; doi:10.1371/journal.pcbi.1008218)
Supplement: S2 Fig — The Pearson correlation coefficients between expression values of k-hop gene pairs from PPI network are shown as box plots. The Pearson correlation coefficients of different hops are shown in each column. (PDF) [file pcbi.1008218.s002.pdf]

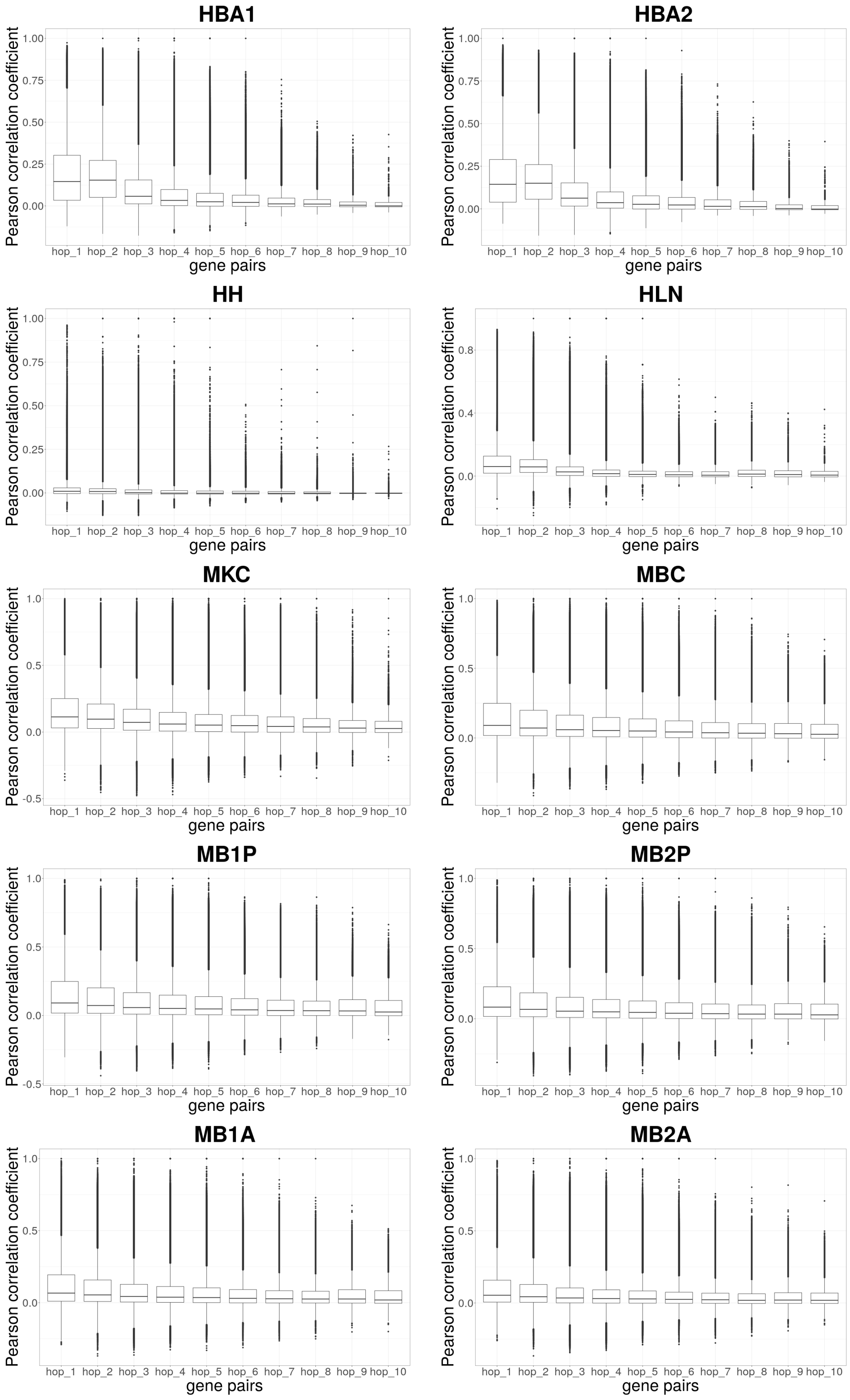

PPI co-expression analysis. The Pearson correlation coefficients between expression values of k-hop gene pairs from PPI network are shown as box plots. The Pearson correlation coefficients of different hop is shown in each column.
