# Supplementary material for: Imputation of spatially-resolved transcriptomes by graph-regularized tensor completion
Source: PLoS Comput Biol. 2021 Apr 7;17(4):e1008218. doi: 10.1371/journal.pcbi.1008218 (PMC8055040; doi:10.1371/journal.pcbi.1008218)
Supplement: S4 Fig — The performances on the imputations of each gene are shown as box plots. The MAPE of every gene slice is denoted by one dot. The performance of each method is shown in each colored box plot. (PDF) [file pcbi.1008218.s004.pdf]

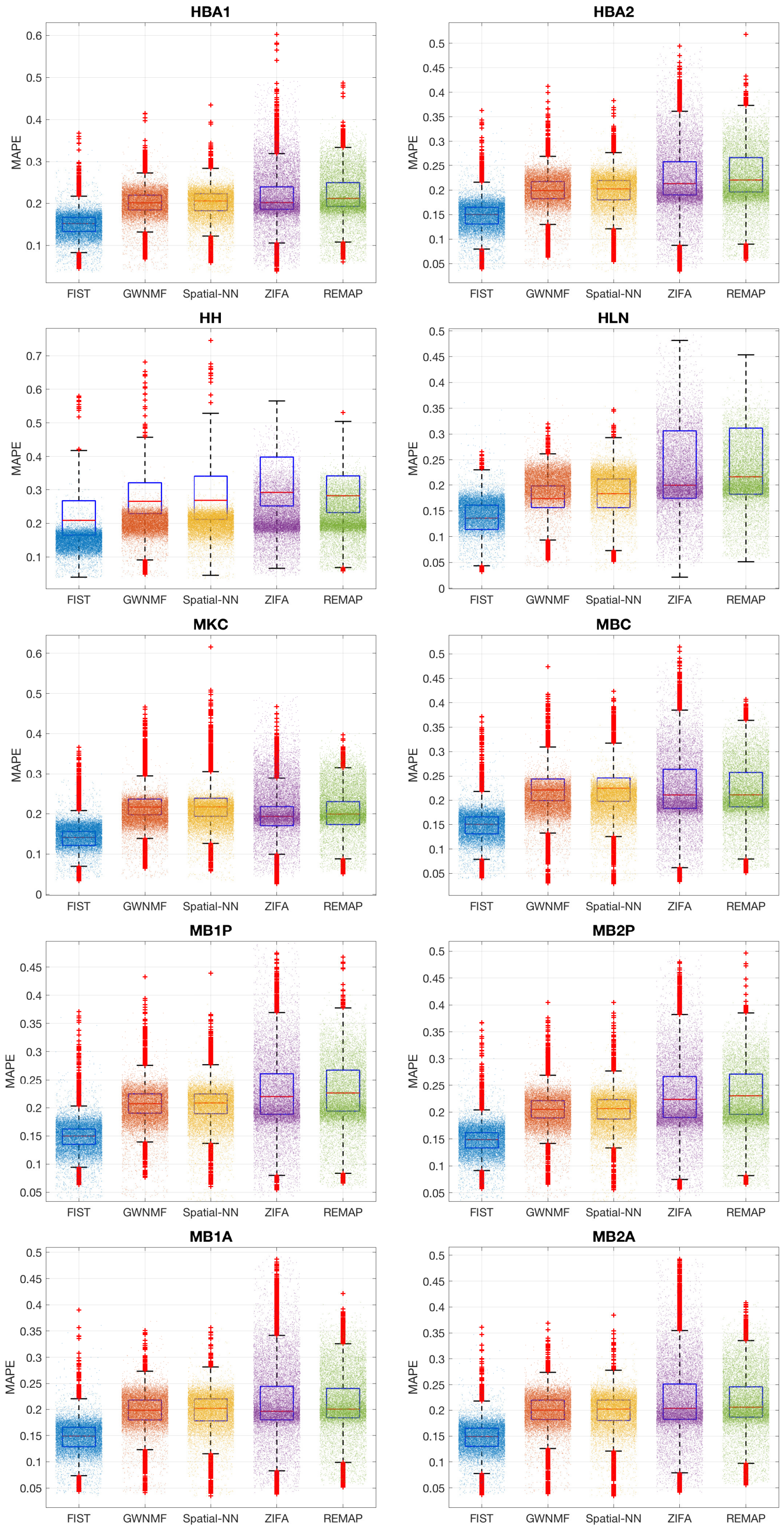

Gene-wise imputation performance by MAPE. The performances on the imputations of each gene are shown as box plots. The MAPE of every gene slice is denoted by one dot. The performance of each method is shown in each colored box plot.
