# Supplementary material for: Imputation of spatially-resolved transcriptomes by graph-regularized tensor completion
Source: PLoS Comput Biol. 2021 Apr 7;17(4):e1008218. doi: 10.1371/journal.pcbi.1008218 (PMC8055040; doi:10.1371/journal.pcbi.1008218)
Supplement: S1 File — (PDF) [file pcbi.1008218.s008.pdf]

S1 File. Convergence of FIST.

We follow the convergence analysis in our previous work Li et al. 2019 to show that FIST can converge under the updating rules in Equations (11)-(13).

As the objective function $\mathcal{J}$ in Equation (3) is bounded from below by zero, we can prove the convergence of FIST by showing that $\mathcal{J}$ is non-increasing under each of the updating rules in Equations (11)-(13). Here, we only show that $\mathcal{J}$ is non-increasing under Equation (11). The proof is directly applicable to Equations (12) and (13).

We first expand the derivative in Equation (6) as

$$\frac{\partial \mathcal{J}}{\partial \hat{A}_p} = -X_1 - \hat{A}_p X_2 - W_p \hat{A}_p X_3 + X_4 + \hat{A}_p X_5 + D_p \hat{A}_p X_3,$$

where $X_1 = (\mathcal{M}_{(1)} \circledast \mathcal{T}_{(1)})(\hat{A}_x \odot \hat{A}_y)$, $X_2 = \lambda(\Phi_x \circledast \Theta_y^W + \Phi_y \circledast \Theta_x^W)$, $X_3 = \lambda(\Phi_x \circledast \Phi_y)$, $X_4 = (\mathcal{M}_{(1)} \circledast \hat{\mathcal{T}}_{(1)})(\hat{A}_x \odot \hat{A}_y)$, and $X_5 = \lambda(\Phi_x \circledast \Theta_y^D + \Phi_y \circledast \Theta_x^D)$.

Theorem 1. *Lee and Seung, 2001: A function $\mathcal{J}(h)$ is non-increasing under the update $h^* \leftarrow \arg\min_h G(h, \tilde{h})$ if $G(h, \tilde{h})$ is an auxiliary function for $\mathcal{J}(h)$, such that the following conditions are satisfied:*

$$G(h, \tilde{h}) \geq \mathcal{J}(h), \quad G(h, h) = \mathcal{J}(h).$$

Based on Theorem 1, $\mathcal{J}$ is non-increasing under the update in Equation (11) if it is an update of one proper *auxiliary function* of $\mathcal{J}(\hat{A}_p)$, which is defined in Theorem 2.

Theorem 2. *The following function*

$$G([\hat{A}_p]_{a,b}, [\tilde{A}_p]_{a,b}) = \mathcal{J}([\tilde{A}_p]_{a,b}) + \mathcal{J}'([\tilde{A}_p]_{a,b})([\hat{A}_p - \tilde{A}_p]_{a,b}) + \frac{[X_4 + \tilde{A}_p X_5 + D_p \tilde{A}_p X_3]_{a,b}}{2[\tilde{A}_p]_{a,b}}([\hat{A}_p - \tilde{A}_p]_{a,b})^2 \quad (1)$$

is an auxiliary function of $\mathcal{J}([\hat{A}_p]_{a,b})$ and has its global minimum.

Proof: First, it is obvious that $G([\hat{A}_p]_{a,b}, [\hat{A}_p]_{a,b}) = \mathcal{J}([\hat{A}_p]_{a,b})$. To show $G([\hat{A}_p]_{a,b}, [\tilde{A}_p]_{a,b}) \geq \mathcal{J}([\hat{A}_p]_{a,b})$ we obtain the second-order Taylor expansion of $\mathcal{J}([\hat{A}_p]_{a,b})$ at the point $[\tilde{A}_p]_{a,b}$ as

$$\mathcal{J}([\hat{A}_p]_{a,b}) = \mathcal{J}([\tilde{A}_p]_{a,b}) + \mathcal{J}'([\tilde{A}_p]_{a,b})([\hat{A}_p]_{a,b} - [\tilde{A}_p]_{a,b}) + \frac{1}{2}\mathcal{J}''([\tilde{A}_p]_{a,b})([\hat{A}_p]_{a,b} - [\tilde{A}_p]_{a,b})^2,$$

with the second-order derivative given below:

$$\mathcal{J}''([\tilde{A}_p]_{a,b}) = -[X_2]_{b,b} - [W_p]_{a,a}[X_3]_{b,b} + [X_5]_{b,b} + [D_p]_{a,a}[X_3]_{b,b}$$

Thus, the inequality $G([\hat{A}_p]_{a,b}, [\tilde{A}_p]_{a,b}) \geq \mathcal{J}([\hat{A}_p]_{a,b})$ holds if

$$\frac{[X_4 + \tilde{A}_p X_5 + D_p \tilde{A}_p X_3]_{a,b}}{[\tilde{A}_p]_{a,b}} \geq \mathcal{J}''([\tilde{A}_p]_{a,b}),$$

which can be demonstrated by the facts that

$$[D_p \tilde{A}_p X_3]_{a,b} = \sum_{l,m} [D_p]_{a,l} [\tilde{A}_p]_{l,m} [X_3]_{m,b} \geq [D_p]_{a,a} [X_3]_{b,b} [\tilde{A}_p]_{ab},$$

and $[\tilde{A}_p X_5]_{a,b} = \sum_{l} [\tilde{A}_p]_{a,l} [X_5]_{l,b} \geq [X_5]_{b,b} [\tilde{A}_p]_{a,b}$. (End of Proof)

As the *auxiliary function* $G([\hat{A}_p]_{a,b}, [\tilde{A}_p]_{a,b})$ in Equation (1) is a quadratic function on variable $[\hat{A}_p]_{a,b}$, its minimum can be easily obtained in a closed-form as

$$\begin{aligned}
[\hat{A}_p]^*_{a,b} &= \arg\min_{[\hat{A}_p]_{a,b}} G([\hat{A}_p]_{a,b}, [\tilde{A}_p]_{a,b}) \\
&= \frac{[\tilde{A}_p]_{a,b}[X_1 + \tilde{A}_p X_2 + W_p \tilde{A}_p X_3]_{a,b}}{[X_4 + \tilde{A}_p X_5 + D_p \tilde{A}_p X_3]_{a,b}},
\end{aligned}$$

which leads to the updating rule in Equation (11).

To analyze the optimality of the fixed point after convergence, we first define $\{\Lambda_p \in \mathbb{R}^{n_p \times r}, \Lambda_x \in \mathbb{R}^{n_x \times r}, \Lambda_y \in \mathbb{R}^{n_y \times r}\}$ to be the matrices of Lagrange multipliers with the Lagrange function

$$\mathcal{L} = \mathcal{J} - \sum_{i \in \{p,x,y\}} \mathrm{tr}(\Lambda_i \hat{A}_i^T).$$

Setting $\frac{\partial \mathcal{L}}{\partial \hat{A}_p}$ to be zero, we obtain $\Lambda_p = \frac{\partial \mathcal{J}}{\partial \hat{A}_p}$. Furthermore, when $A^{(i)}$ is a fixed point under the updating in Equation (**??**) we have

$$[-X_1 - \hat{A}_p X_2 - W_p \hat{A}_p X_3 + X_4 + \hat{A}_p X_5 + D_p \hat{A}_p X_3]_{a,b}[\hat{A}_p]_{a,b} = 0,$$

which implies the KKT complementary slackness condition $[\Lambda_p]_{a,b}[A_p]_{ab} = 0$ is satisfied.
