# Supplementary material for: Imputation of spatially-resolved transcriptomes by graph-regularized tensor completion
Source: PLoS Comput Biol. 2021 Apr 7;17(4):e1008218. doi: 10.1371/journal.pcbi.1008218 (PMC8055040; doi:10.1371/journal.pcbi.1008218)
Supplement: Attachment — Submitted filename: response_to_reviewers.pdf [file pcbi.1008218.s011.pdf]

UNIVERSITY OF MINNESOTA

Twin Cities Campus

Department of Computer Science and Engineering
College of Science and Engineering

4-192 Keller Hall
200 Union Street S.E.
Minneapolis, MN 55455-0159

612-625-4002
Fax: 612-625-0572
http://www.cs.umn.edu

Summary of Revisions

Title: Imputation of Spatially-resolved Transcriptomes by Graph-regularized Tensor Completion

Authors: Zhuliu Li, Tianci Song, Jeongsik Yong, and Rui Kuang

We sincerely thank the reviewers and the editorial board for the very constructive comments on our previous submission for improving our research work. We made substantial revisions in this resubmission to respond to the comments. Below is a summary of the major changes that we made to address the key points in the review, followed by the responses to the specific comments from each reviewer.

- To clarify and articulate our formulation of imputation, we added a new spot-wise imputation experiment in addition to the gene-wise imputation. In the new experiment, we chose 4-fold of the non-empty spatial spots for training and validation, and hold out the rest 1-fold non-empty spatial spots as test data, assuming all the gene expressions over the test spots are missing. The new results are shown in Figure 2, Figure 4 and S1 Table, and discussed on pages 11 and 14. The new results are very consistent with our gene-wise imputation experiments, and support the conclusions in this study.

- In addition, to show that the imputation of gene expressions over some spots with failed RNA capture is a specific imputation problem for spatial genomics data, we also showed a few such examples in S1 Figure, which are also discussed on page 2 as one key motivation of our work.

- To demonstrate that FIST is broadly applicable to impute the spatial gene expression data generated with different platforms, we performed additional experiments on the spatial transcriptomics datasets from 3 replicates of mouse tissue (olfactory bulb) provided by Ståhl et al. (2016). The new results are shown in Figure 8 and 9, and discussed on pages 18 and 19. We observe that FIST clearly outperforms all the baselines with lower MAE and MAPE, and larger R^2 in all the 3 replicates, which is consistent with the observation in Figure 2 and Figure 3 using the 10x Visium spatial genomics data.

- We performed additional analysis to examine the relation between the connectivity in PPI network and the co-expression in spatial locations among the genes in the 10 different 10x Genomics Visium spatial genomics datasets used in this study. The results of this analysis are shown in S2 Figure. We observed higher co-expressions between the genes that are connected with less hops in the PPI, which supports one key hypothesis in FIST: genes' connectivities in PPI network highly correlate with their co-expressions.

- To improve the clarity of presentation, 1) we included the original H&E image of the Mouse Kidney Section in Figure 7 (was Figure 6), and marked the three biological regions (Cortex,

ISOM and OSOM) on the H&E image; 2) we moved Figure 3 to Supplementary as S3 Figure; 3) we reformatted the plot of the factor matrices in Figure 1 (C).

- We made substantial changes in our GitHub repository to help readers reproduce our results. The key changes are summarized as: 1) we made all the datasets and key results available to download through a link provided in the README.md file; 2) we provided complete code scripts in the repo and step-by-step instructions in the README.md for readers to run the cross-validation experiments in the paper; 3) we provided code scripts and instructions to convert the raw spatial transcriptomic datasets into regular tensors; 4) we provided the code scripts to display the key results reported in the paper.
- We made substantial changes of our software package in our GitHub repository (`https://github.com/kuanglab/FIST`) to reproduce all the experimental results and usability.

We highlighted all the revised text in the manuscript. Below are the responses to each comment.

Responses to Reviewer: 1 (Technical Review)

To improve the reproducibility of all the results and better usability by users of our GitHub package, we made the following key changes in our GitHub repository at `https://github.com/kuanglab/FIST`,

- We made all the datasets and key results available to be downloaded through a link provided in the README.md file.
- We provided complete scripts in the repo and step-by-step instructions in the README.md for readers to run the cross-validation experiments in the paper.
- We provided the scripts and instructions to convert the raw spatial transcriptomic datasets into regular tensor data as inputs for FIST.
- We provided the scripts to display the key results reported in the paper.

Responses to Reviewer: 2

This paper proposes a computational method, termed FIST, for the analysis of high-throughput spatial-transcriptomics RNA sequencing (sptRNAseq) data. sptRNAseq data suffers from high drop-out rate, preventing the correct characterization of gene transcriptional programs. To overcome this issue, the authors propose an approach based on graph regularized Tensor-completion. This method not only harnesses the spatial and transcriptomic information in the data, but also uses a protein-protein interaction network as a prior, to relate genes with similar functions, and a spatial graph. Even more, the authors validate their methods on widely available datasets and explore how the p-p interaction network improve the accuracy of their imputation predictions. The code is available on GitHub and can be effortlessly download.

The proposed framework is elegant and well described and based on the experiments performed seems to be able to have a higher accuracy compared to other approaches. A nice feature of the approach is that it computes a compressed representation of the data given by its tensor polyadic canonical decomposition. These points fulfill the requirements of originality and innovation. In addition, SptRNAseq is a technology developed 3 years ago and a commercial platform was released just six months ago. This makes the method attractive to the community but puts an emphasis on making it easy-to-use for users, satisfying the requirement of importance in the field and methodological insight. I would recommend publication of the method after my concerns are addressed (and some minor points) that are dampening my enthusiasm (to strengthen the points of a rigorous methodology with substantial evidence for its conclusions).

Response:
We appreciate the positive comments provided by the reviewer. We carefully addressed the major and minor comments are follows.

1. The method is evaluated using 5 fold cross-validation on datasets from the 10x Visium platform. The method is contrasted against methods develop to process scRNA-seq data (ZIFA and REMAP) or artificially generated methods that use spatial information. A quick search on google returned methods that are specifically developed to analyze sptRNAseq (like "joint model of unpaired data from scRNA-seq and spatial transcriptomics for imputing missing gene expression measurements", which I am not an author in). It would be great that the authors add a comparison against a method specifically develop to impute missing spatial data. In addition, the authors should test the method on one more technology (not just 10x) to show broad applicability (again, publications in journals show databases that compile spatial transcriptomic data from different technologies).

   Response:
   Thanks for the comment on the baseline methods, and suggesting to test one additional spatial dataset generated with different technology.

   - On page 18-19 of this resubmission, we performed additional experiments on the spatial transcriptomics datasets of lower resolutions from 3 replicates of mouse tissue (olfactory bulb) provided by Ståhl et al. (2016) using earlier spatial transcriptomics technologies. From the new results shown in the Figure 8 and Figure 9, we observe that FIST clearly outperforms all the baselines with lower MAE and MAPE, and larger R^2 in all the 3 replicates, which is consistent with the observation in Figure 2 and Figure 3 using the 10x Genomics data. The results suggest that FIST is a general method applicable for various spatial transcriptomics datasets, and achieves superior performance by modeling the spatial data as tensors, and including the prior knowledge as CPG regularization.
   - Thanks for reminding us the work in Lopez et al. (2019). The work in Lopez et al. (2019) is intended for imputing genes that are not profiled in the spatial gene expression

data (such as those collected by smFISH) that only profile a subset of genes rather than the whole transcriptome using single-cell RNA sequencing (scRNA-seq) data. Thus, the method is not applicable to the transcriptome-wide 10X Visium spatial genomic datasets in our experiments since we don't have matched scRNA-seq data. We also tested the package provided by the authors, and found out that there also could be scalability issues in training the graphical model for whole-transciprome data. Therefore, we did not include Lopez et al. (2019) in our baselines. Instead, we add a discussion of the method in Lopez et al. (2019) in the discussion section as a future direction for our work.

2. The Results Section "FIST imputations recover spatial patterns enriched by highly relevant functional terms" falls short to demonstrate that true biological insights can be extracted with the method. It is difficult to conclude much from figure 6 and interpret why the shape of the computed clusters is biologically relevant. The figure has to be made more informative, maybe adding GO terms or additional results should be added. In addition, enriched GO terms calculation (figure 5) should be study more carefully. Are these GO terms relevant or is the algorithm producing spurious results? More biologically meaningful results should be added to strengthen the interest in the paper.

   Response:
   Thanks for pointing out the clarity issues in Figure 6 and its connection with Table 3. In the resubmission, the Figure 6 now becomes Figure 7.

   - To improve the clarity, we included the original H&E image of the Mouse Kidney Section in Figure 7, and marked the three biological regions (Cortex, ISOM and OSOM) on it. More details of these three regions are explained in the NIH website [1]. Due to the interesting observation that the cluster centers obtained by FIST are similar in shapes as the Cortex, ISOM and OSOM in mouse kidney, we feel it is worthwhile to further investigate functions of the genes in these clusters.
   - To validate our assumption that each cluster in Figure 7 is functionally related to each of the three biological regions in mouse kidney, we performed functional enrichment analysis to the genes in each cluster. The enriched GO terms in Table 3 demonstrate that each gene cluster indeed functionally relates to each biological region with high significance (small p-values). We also report the complete list of enriched GO terms in supplementary Table 2. It is clear that there is no spurious results since there are only a small number of enriched GO terms with highly specific functions across the clusters. The evidences of relations between the GO terms and biological regions are all reported in literature review in last paragraph of this section. Therefore, based on our visualizations and enrichment analysis, we believe that there is strong evidence that FIST is capable of recovering spatial gene expression patterns with highly relevant functional characteristics among the genes in the spatial regions.

3. The method is available on GitHub and it is easily downloadable. However, there is no instructions as to how to analyze a new dataset. The authors should add a section in the GitHub repo with instructions for users to be able to analyze their own data.

   Response:
   Thanks for the comment on improving our GitHub repository. The prerequisite for the application of FIST to a new dataset is to convert the raw spatial transcriptomic data into

[1] `https://ntp.niehs.nih.gov/nnl/urinary/kidney/index.htm`

a tensor. However, different datasets might need different preprocessing strategies. To help readers understand our data preprocessing for the experiments, we provided two R scripts **Convert2Tensor_Visium.R** and **Convert2Tensor_ST.R** with step-by-step instructions for how to convert the raw spatial transcriptomic datasets from 10x Genomics and Ståhl et al. (2016) to tensors. In the README file, we also provided detailed instructions for how to perform the spot-wise and gene-wise evaluations on FIST, starting from the raw datasets.

Minor Mathematical Issues:

1. It would be great if the authors specify how n_x, n_y, n_p are considered from the data. Am I right to believe that n_x and n_y would be dependent of the number of spots and that they will be discretize according to the spots per se. This should be made clearer.

   Response:
   Many thanks for the comment. Indeed, the spots are arranged in a perfect grid in earlier spatial transcriptomic arrays to map the coordinates (n_x,n_y). In the Visium array slide, the spots are arranged in a hivegrid. We actually shifted the odd-numbered rows by half of a spot as an approximated grid for tensor modeling. This approximation does not introduce any assumption other then the convenient arrangement of the spots into the tensor and the spatial graph. We added the clarification in the section "Preparation of spatial gene expression datasets" on page 10.

2. Would this method be useful to harness information from different datasets? How do you know that the tissue is aligned among samples?

   Response:
   Thanks for the suggestion. We also realized it might be very challenging to integrate multiple samples (such as biological replicates) due to the difficulty of aligning the grids across arrays. Currently, FIST only solves within array imputation. It is possible to have a preprocessing step to align the grids and then apply a 4-way tensor for the problem or design a multi-task learning problem to integrate multiple 3-way tensors. We do not have a solid solution yet and plan to leave this as one of our future directions as we discussed in the Discussion of this resubmission.

3. Why do the authors introduce $\mathcal{M}$ and observed entries of $\mathcal{T}_{i,j,k}$. Why would the entries not being observed? If this is done for cases in which some spatial coordinates are not observed, or genes, this should be made clear. It was confusing to me to understand if they were going to consider the zeroes in the data as unobserved or unobserved data is given by the experimentalist. Is dropout unobserved or missing, how do you choose $\mathcal{M}$?

   Response:
   Thanks for the excellent point on how to design the tensor $\mathcal{M}$.

   - Our approach is to model the imputation problem as tensor completion by using known (or more reliable) entries to estimate the other missing (or uncertain) entries. This tensor modeling is flexible in imputing different types of missing (or poorly measured) data such as 1) some spots that entirely failed fixing and permeabilizing RNAs or 2) those genes with relatively lower RNA capture efficiency due to the sequence compositions.
   - To clarify and articulate the imputation problems, we performed both 1) spot-wise imputation (newly added) and 2) gene-wise imputation experiments. As described in the section "Evaluations and performance measures", for both experiments, we performed

cross-validation on the non-zeros in the gene expression tensor $\mathcal{T}$ to evaluate the performance. The training entries in $\mathcal{T}$ were indicated as 1s in $\mathcal{M}$, versus the test entries in $\mathcal{T}$ were indicated as 0s in $\mathcal{M}$ and treated as missing data.

- In the resubmission, we also clarified the imputation problem in the abstract, author summary and the introduction (page 2-3). In particular, the imputation of gene expressions over some spots with failed RNA capture is a specific problem for spatial genomics data and we showed a few such examples in S1 Figure. In our ongoing project with plant biologists, we also observed similar difficulties in preparing tissue sections and fixing/permabilizing RNAs. Thus, we believe FIST can be a useful tool for such imputation tasks for spatial transcriptomics data.

4. How complete is the PPI network? Can this be biasing the inferential procedure due to missing or incorrect links in this network?

   Response:
   Thanks for the comment on the quality of the PPI network and its contribution to the imputation task.

   As described at the end of the section "Preparation of spatial gene expression datasets" on page 10, we downloaded the full Homo sapiens and Mus musculus protein-protein interactions (PPI) networks from BioGRID 3.5. The BioGRID PPI networks collect a comprehensive set of PPIs from experimental data and related literature. Our problem formulation utilizes the topological structure of the PPI networks with a graph Laplacian regularization rather than only direct neighbors and thus, is relatively robust when some PPIs are missing. Empirically, we also observed that the performance of the imputation of the genes does not depend on the number of neighbors that a gene has in the PPI. In Figure 5, we also show the results on permuted graphs. It is also clear that the original networks contain useful information to improve the imputation performance.

   In the resubmission, we also performed additional analysis to examine the relation between the connectivity in PPI network and the co-expression in spatial locations among the genes in the 10 different 10x Genomics Visium spatial genomics datasets used in this study. The results of this analysis are shown in S2 Figure. We observed higher co-expressions between the genes that are connected with less hops in the PPI, which supports one key hypothesis in FIST: genes' connectivities in PPI network highly correlate with their co-expressions.

   Thus, we believe the BioGrid PPI networks are playing a useful role in our model to improve the imputation.

5. "We then use the Cartesian product $\mathcal{G}(x, y, p) = (V, E)$ of the three individual graphs G_x, G_y and G_p to regularize the elements in $\hat{\mathcal{T}}$, where $|V| = n_x n_y n_p$." Can you discuss more this huge network? Can you store it? is it sparse? How many edges are there?

   Response:
   Thanks for the comment on the scalability of FIST regarding the Cartesian product graph (CPG) regularization. One benefit of FIST algorithm is the computational efficiency without requiring explicit construction or computation of the Cartesian product graph. With efficient tensor computations in Equations (7)-(10) described in Section "FIST Algorithm" on page 7, the tensor calculus for FIST is broken down into the computations on the individual graphs and the sparse tensors. Therefore, we do not need to compute the full CPG even though the objective function contains the CPG regularization. In the same section of the resubmission, we also included the space complexity of FIST together with the time complexity to justify that FIST is a scalable methods without need of computation with the full CPG.

6. This might be a trivial result but can you clarify it. "Denoting the adjacency, degree and graph Laplacian matrices of graph G_i as W_i, D_i and $L_i = D_i - W_i$." I would have believed that D is the degree matrix but here, is it W?

   Response:
   Sorry for the confusion in the previous submission. To be more clear, in the resubmission we changed this statement to "For a graph $G_i = (V_i, E_i)$ where $i \in \{x, y, p\}$, we denote its adjacency matrix as W_i, degree matrix as D_i, and graph Laplacian matrix as $L_i = D_i - W_i$".

7. The FIST algorithm is clear, but it refers for its calculation to many other results. While reading it, I thought this should be shortened but I leave it to the authors to improve its presentation. This part should be made more accessible to non-specialists.

   Response:
   Thanks for the suggestion. To improve the readability of the FIST algorithm presented in Algorithm 1, we also summarized the algorithm by more explanations of each line in the end of Section "FIST Algorithm" as "FIST takes the incomplete spatial gene expression tensor $\mathcal{T}$, PPI network and spatial chain graphs as input (line 1-2 in Algorithm 1), and outputs the inferred CPD representation of the complete spatial gene expression tensor $\hat{\mathcal{T}}$ (line 9 in Algorithm 1), via solving the optimization problem defined in Proposition 1 with the multiplicative updating rule (line 5-7 in Algorithm 1) based on the tensor calculus in Equations (7)-(10)."

8. Is the value of lambda transferable? Among data sets? Do you need to train the algorithm every time? If the authors explore different technologies, they can show that maybe, a value of lambda is unique and need not to be retrained.

   Response:
   Thanks for the comment on the hyper-parameter selection. As for most the existing machine learning methods, we suggest users to select the hyper-parameter λ on the held-out validation set. In our experiments, we select λ from the pool $\{0, 0.01, 0.1, 1\}$ as mentioned on page 9 through cross-validation. Interestingly, we do observe from the Figure 4 and Figure 9 in the resubmission that $\lambda = 0.1$ seems to be an optimal value which is transferable among the 10 tissue sections from the 10x Genomics and the 3 mouse tissue replicates from Ståhl et al. (2016). Even so, we still recommend users to select λ on a validation set to optimize the performance of FIST while is applied to a new dataset.

9. Are the authors bothered by the idea of finding a local optimum of a non-convex algorithm? Do we want the global optima?

   Response:
   Thanks for the comment on the optimization in FIST.

   - As the goal of this study is for tensor completion, it is not necessary to find the unique tensor factor matrices $\hat{A}_p$, $\hat{A}_x$, and $\hat{A}_y$ (global minimum) since different factor matrices can lead to the similar CPD reconstruction $\hat{\mathcal{T}} = [\![\hat{A}_p, \hat{A}_y, \hat{A}_x]\!]$. We have also observed from the experiments that FIST with different randomly initialized factor matrices lead to very similar imputation results as expected.
   - Moreover, the non-convex optimization problem presented in Proposition 1 can be easily converted to a convex problem by dropping the equality constraint $\hat{\mathcal{T}} = [\![\hat{A}_p, \hat{A}_y, \hat{A}_x]\!]$. However, in such case, the time and space complexities become inevitably high as computing the full dense tensor and Cartesian product graph becomes necessary.

Minor Mathematical Issues:

1. The discussion section begins with the sentence: "In this study, we proved that tensor is a natural representation of the multidimensional structure in spatially-resolved gene expression data mapped by the 2D spatial array". I do not think that *prove* is the right word here. This type of data can be represented as a tensor and provably by many other mathematical constructs.

   Response:
   Thanks for the comment, in the resubmission we changed this statement to "In this study, we proposed to use tensor to represent the multidimensional structure in spatially-resolved gene expression data mapped by the 2D spatial array."

2. A sentence reads: "Apparently, it becomes computationally expensive and often infeasible to compute or store a dense tensor, especially in high spatial resolutions with millions of spots.". Again, *apparently* is not the right word here. It is or it is not computationally expensive to store the tensor.

   Response:
   Thanks for the comment. In the resubmission, we changed this statement to "However, it is computationally expensive and often infeasible to compute or store a dense tensor $\hat{\mathcal{T}}$, especially in high spatial resolutions with millions of spots."

3. Equation 2 is missing a parenthesis.

   Response:
   Thanks for finding this typo. We corrected in the resubmission.

4. A sentence reads: "we also further tested that FIST is also applicable and scalable to high-resolution spatial transcriptomics datasets with millions of spots in the preliminary work in a follow-up study". If the authors tested the method on a denser dataset, they should include this result. If not, this sentence sounds anecdotal and not scientifically sound, and the sentence should be removed or added as a discussion point for future work.

   Response:
   Thanks for the suggestion. Indeed, it is tricky to formulate the imputation problem in HDST datasets due to the high-resolution of the data. None of the methods including FIST works reasonably well on HDST datasets. We changed our statements and mentioned the work as our future work in the "Discussions" section.

Responses to Reviewer: 3

The paper presents an approach to imputation of gene expression in spatial transcriptomics data using graph-regularised tensor completion. This is an interesting class of model for spatial data and I can imagine that it could be useful in smoothing data or in other data analysis tasks, e.g. smoothing followed by clustering may well be a good approach. I can also believe that the method performs well in the synthetic benchmarks where data are removed (at random I think?) and then imputed, compared to the other models considered here. However, it was not clear to me exactly how it should be used in real data. Are the authors suggesting that all zeros should be imputed with non-zero values? Is this what is done (I was not sure) and is that justified? The zeros are not missing-at-random data, they are data that happens to be zero usually because expression levels are low and read-depth is limited. Replacing them with non-zero values seems to me to be more akin to smoothing than to imputation. It is therefore not clear to me that this is justified. Smoothing

may be useful prior to modelling (e.g. clustering) but that is not the same as imputation which should really only be used to deal with missing data, not low counts data. I'm afraid I therefore don't agree with the way the method is presented as an imputation approach. I have two specific major concerns:

Response:

Thanks for the comments and pointing out the difference between imputation and smoothing/normalization. This is definitely a valid point. Our approach is to model the imputation problem as tensor completion by using known (or more reliable) entries to estimate the other missing (or uncertain) entries. This tensor modeling is flexible in imputing different types of missing (or poorly measured) data such as some spots that entirely failed fixing and permeabilizing RNAs or those genes with relatively lower RNA capture efficiency due to the sequence compositions. We now clarified the imputation problem in the abstract, author summary and the introduction (page 2-3). In particular, the imputation of gene expressions over some spots with failed RNA capture is a specific problem for spatial genomics data and we showed a few such examples in S1 Figure. In our ongoing project with plant biologists, we also observed similar difficulties in preparing tissue sections and fixing/permabilizing RNAs. Thus, we believe FIST can be a useful tool for such imputation tasks for spatial transcriptomics data.

To clarify and articulate the imputation problems, we added a new spot-wise imputation experiment in addition to the gene-wise imputation. In the spot-wise imputation experiment, we chose 4-fold of the non-empty spatial spots for training and validation, and hold out the rest 1-fold non-empty spatial spots as test data, assuming all the gene expressions over the test spots are missing. We described the new imputation experiments on pages 11. The new results are shown in Figure 2, Figure 4 and S1 Table, and discussed on page 11 and 14. The new results are very consistent with our gene-wise imputation experiments and support the conclusions in this study.

Major comments:

1. It is not clear in modern UMI-normalised data whether (or when) zeros should be considered artefacts to be corrected/imputed since they may simply reflect genuinely weak expression. If the expression is weak then imputation should not be used and will smooth out genuine signal in the data. If genes are weakly expressed then counts can be very low and zeros simply reflect read depth limitations. For example, in the case of single-cell RNA-Seq data recent work shows that UMI-normalised data is not highly zero-inflated [1,2,3] and this was really only a problem on older datasets without UMI normalisation where amplification artefacts were harder to remove. I imagine the same holds true for UMI-normalised spatial transcriptomics data, in which case imputation of zero-valued genes is not justified. The citations in the introduction discussing modelling zero-inflation (Refs [22, 25, 26. 27] in the paper) predate these more recent works and often model zero-inflation as an artefact to be corrected for rather than simply part of a standard measurement model such as a negative binomial distribution over counts.

   Response:

   Thanks for the comments. We completely agree that, in our previous submission, it was misleading to mention imputing dropouts as the motivation of our imputation tasks on spatial transcrptomics data. As we explained in the response to the general comments by the reviewer, we clarified this point in this revision and added more experiments to improve our work. Thanks again for the excellent suggestion.

   We also agree that high sparseness does not necessarily imply zero-inflation depending on the assumed underlying distribution. We do want to point out that our approach is not based on the assumption of any zero-inflated distributions. Our main assumptions are on the spatial structure among the spots and the functional relations among the genes, both of

which are introduced to overcome the challenges from the high sparsity of the sptRNA-seq data regardless if the underlying distribution is zero-inflated or not.

In addition, droplet-based platform followed by UMI tag sequencing has relatively low RNA capturing efficiency compared with traditional LCM or FACS captures. Thus, droplet + UMI data is typically sparser than LCM/FACS + RNAseq data. The lowly expressed genes reported by different single-cell capture/sequencing platforms or bulk RNAseq can be quite different. We do agree that further investigation of the types of dropouts in scRNA-seq and sptRNA-seq is necessary in future work although it is less relevant to the work in this manuscript.

2. Another major concern I have it that PPI data is used to regularise genes on the basis that interacting genes are more likely to be correlated in gene expression. I was wondering how good is this assumption since it seems very easy to check with data. Another approach would be to use previous expression datasets to assess co-expression, for example, since expression data are so plentiful. Is PPI the best choice for determining whether genes are likely correlated? This could be tested on observed expression data, e.g. how much co-expression is explained by PPI data?

   Response:
   Thanks for the excellent point. We added an experiment to check the relation between gene connectivities in PPI and the gene co-expressions across the spots. The results are shown in S2 Figure. There is a significant difference in the strength of co-expressions between connected gene pairs and unconnected gene pairs. We now mention this observation as a motivation in the main text on page 6.

   Note that rather than explaining the observed co-expressions, PPI network actually provides functional relations among the genes beyond the observed co-expressions, which is particularly useful for sparsely expressed genes exhibiting relatively lower co-expressions with other genes as we show in Figure 5(A).

References

[1] Choi, K., Chen, Y., Skelly, D. A., & Churchill, G. A. (2020). Bayesian model selection reveals biological origins of zero inflation in single-cell transcriptomics. bioRxiv.

[2] Svensson, V. (2020). Droplet scRNA-seq is not zero-inflated. Nature Biotechnology, 38(2), 147-150.

[3] Svensson, V. (2020). Sarkar, A. K., & Stephens, M. (2020). Separating measurement and expression models clarifies confusion in single cell RNA-seq analysis. BioRxiv.
