# Supplementary material for: Imputation of spatially-resolved transcriptomes by graph-regularized tensor completion
Source: PLoS Comput Biol. 2021 Apr 7;17(4):e1008218. doi: 10.1371/journal.pcbi.1008218 (PMC8055040; doi:10.1371/journal.pcbi.1008218)
Supplement: Attachment — Submitted filename: Response_to_Reviewers.pdf [file pcbi.1008218.s013.pdf]

University of Minnesota

*Twin Cities Campus*

***Department of Computer Science and Engineering***
*College of Science and Engineering*

*4-192 Keller Hall*
*200 Union Street S.E.*
*Minneapolis, MN 55455-0159*

*612-625-4002*
*Fax: 612-625-0572*
*http://www.cs.umn.edu*

# Summary of Revisions

## Title: Imputation of Spatially-resolved Transcriptomes by Graph-regularized Tensor Completion

**Authors: Zhuliu Li, Tianci Song, Jeongsik Yong, and Rui Kuang**

We sincerely thank the reviewers and the editorial board for appreciating our work, and proving constructive comments to improve the presentation of our work.

**No revision is needed for the comments from reviewer 1 and reviewer 3.** Below is our response to reviewer 2's comment which needs the revision: In the abstract it reads: "FIST significantly outperformed several best performing single-cell RNAseq data imputation methods." "several best performing methods" sounds like an oxymoron. There is only one best performing method, not several.

- Thanks for pointing out the inaccurate statement in our abstract. In the revision, we changed this statement to "FIST significantly outperformed the state-of-the-art methods for single-cell RNAseq data imputation". We also highlighted the revised text in the manuscript.
- According to the PLOS formatting guidelines, 1) we moved the proof of convergence to the S1 File in the "Supporting Information" section; 2) we removed the funding information from the "Acknowledgement" section.
