# Supplementary material for: Imputation of spatially-resolved transcriptomes by graph-regularized tensor completion
Source: PLoS Comput Biol. 2021 Apr 7;17(4):e1008218. doi: 10.1371/journal.pcbi.1008218 (PMC8055040; doi:10.1371/journal.pcbi.1008218)
Supplement: Attachment — Submitted filename: review.docx [file pcbi.1008218.s010.docx]

Review: Imputation of Spatially-resolved Transcriptomes by Graph-regularized Tensor Completion.

PLOS Comp Bio criteria for publication:

1. Originality
2. Innovation
3. High importance to researchers in the field
4. Significant biological and/or methodological insight
5. Rigorous methodology
6. Substantial evidence for its conclusions

This paper proposes a computational method, termed FIST, for the analysis of high-throughput spatial-transcriptomics RNA sequencing (sptRNAseq) data. sptRNAseq data suffers from high drop-out rate, preventing the correct characterization of gene transcriptional programs. To overcome this issue, the authors propose an approach based on graph regularized Tensor-completion. This method not only harnesses the spatial and transcriptomic information in the data, but also uses a protein-protein interaction network as a prior, to relate genes with similar functions, and a spatial graph. Even more, the authors validate their methods on widely available datasets and explore how the p-p interaction network improve the accuracy of their imputation predictions. The code is available on github and can be effortlessly download.

The proposed framework is elegant and well described and based on the experiments performed seems to be able to have a higher accuracy compared to other approaches. A nice feature of the approach is that it computes a compressed representation of the data given by its tensor polyadic canonical decomposition. These points fulfill the requirementsof originality and innovation. In addition, SptRNAseq is a technology developed 3 years ago and a commercial platform was released just six months ago. This makes the method attractive to the community but puts an emphasis on making it easy-to-use for users, satisfying the requirement of importance in the field and methodological insight. I would recommend publication of the method after my concerns are addressed (and some minor points) that are dampening my enthusiasm (to stretghten the points of a rigorous methodology with substantial evidence for its conclusions).

1) The method is evaluated using 5 fold cross-validation on datasets from the 10x Visium platform. The method is contrasted against methods develop to process scRNA-seq data (ZiFA and REMAP) or artificially generated methods that use spatial information. A quick search on google returned methods that are specifically developed to analyze sptRNAseq (like “joint model of unpaired data from scRNA-seq and spatial transcriptomics for imputing missing gene expression measurements”, which I am not an author in). It would be great that the authors add a comparison against a method specifically develop to impute missing spatial data. In addition, the authors should test the method on one more technology (not just 10x) to show broad applicability (again, publications in journals show databases that compile spatial transcriptomic data from different technologies).
2) The Results Section “FIST imputations recover spatial patterns enriched by highly relevant functional terms” falls short to demonstrate that true biological insights can be extracted with the method. It is difficult to conclude much from figure 6 and interpret why the shape of the computed clusters is biologically relevant. The figure has to be made more informative, maybe adding GO terms or additional results should be added. In addition, enriched GO terms calculation (figure 5) should be study more carefully. Are these GO terms relevant or is the algorithm producing spurious results? More biologically meaningful results should be added to strengthen the interest in the paper.
3) The method is available on github and it is easily downloadable. However, there is no instructions as to how to analyze a new dataset. The authors should add a section in the github repo with instructions for users to be able to analyze their own data.

Minor Mathematical Issues:

- It would be great if the authors specify how n_x, n_y, n_p are considered from the data. Am I right to believe that n_x and n_y would be dependent of the number of spots and that they will be discretize according to the spots per se. This should be made clearer.
- Would this method be useful to harness information from different datasets? How do you know that the tissue is aligned among samples?
- Why do the authors introduce M and observed entries of t_{i,j,k}. Why would the entries not being observed? If this is done for cases in which some spatial coordinates are not observed, or genes, this should be made clear. It was confusing to me to understand if they were going to consider the zeroes in the data as unobserved or unobserved data is given by the experimentalist. Is dropout unobserved or missing, how do you choose M?
- How complete is the PPI network? Can this be biasing the inferential procedure due to missing or incorrect links in this network?
- “We then use the Cartesian product G(x, y, z) = (V,E) of the three individual graphs Gx, Gy and Gp to regularize the elements in ˆ T , where |V | = nxnynp.”

Can you discuss more this huge network? Can you store it? is it sparse? How many edges are there?

- This might be a trivial result but can you clarify it. “Denoting the adjacency, degree and graph Laplacian matrices of graph Gi as Wi, Di and Li = Di −Wi“. I would have believed that D is the degree matrix but here, is it W?
- The FIST algorithm is clear, but it refers for its calculation to many other results. While reading it, I thought this should be shortened but I leave it to the authors to improve its presentation. This part should be made more accessible to non-specialists.
- Is the value of lambda transferable? Among data sets? Do you need to train the algorithm every time? If the authors explore different technologies, they can show that maybe, a value of lambda is unique and need not to be retrained.
- Are the authors bothered by the idea of finding a local optimum of a non-convex algorithm? Do we want the global optima?

Minor Presentation Issues:

- The discussion section begins with the sentence:” In this study, we proved that tensor is a natural representation of the multidimensional structure in spatially-resolved gene expression data mapped by the 2D spatial array”. I do not think that *prove* is the right word here. This type of data can be represented as a tensor and provably by many other mathematical constructs.
- A sentence reads: “Apparently, it becomes computationally expensive and often infeasible to compute or store a dense tensor, especially in high spatial resolutions with millions of spots.”. Again, *apparently* is not the right word here. It is or it is not computationally expensive to store the tensor.
- Equation 2 is missing a parenthesis.
- A sentence reads: “we also further tested that FIST is also applicable and scalable to high-resolution spatial transcriptomics datasets with millions of spots in the preliminary work in a follow-up study”. If the authors tested the method on a denser dataset, they should include this result. If not, this sentence sounds anecdotal and not scientifically sound, and the sentence should be removed or added as a discussion point for future work.
